# Acetylcholine muscarinic M2 receptor maintains human Schwann-like adipose-derived phenotype in the absence of differentiating medium

**DOI:** 10.1038/s41420-025-02404-0

**Published:** 2025-04-13

**Authors:** Roberta Piovesana, Alessandro Faroni, Ada Maria Tata, Adam J. Reid

**Affiliations:** 1 Department of Biology and Biotechnologies “Charles Darwin”, Sapienza, University of Rome, Rome, Italy; 2 Blond McIndoe Laboratories, Division of Cell Matrix Biology and Regenerative Medicine, School of Biological Sciences, Faculty of Biology, Medicine and Health, The University of Manchester, Manchester Academic Health Science Centre, Manchester, M13 9PT UK; 3 Research Centre of Neurobiology “Daniel Bovet”, Sapienza, University of Rome, Rome, Italy; 4 Department of Plastic Surgery & Burns, Wythenshawe Hospital, Manchester University NHS Foundation Trust, Manchester Academic Health Science Centre, Manchester, M23 9LT UK; 5 Present Address: Groupe de Recherche sur le Système Nerveux Central, Département de Neurosciences, Université de Montréal, Montréal, QC H3T 1J4 Canada

**Keywords:** Schwann cell, Mesenchymal stem cells

## Abstract

Human adipose-derived stem cells (ASCs), differentiated in vitro towards Schwann-like phenotype (hdASCs), have been suggested as an alternative source of Schwann cells (SCs). However, although they seem a good alternative, their differentiation is unstable, losing their SC-like phenotype following growth factor withdrawal. Rat and human SCs and rat dASCs have been characterized for acetylcholine M2 muscarinic receptor subtype that plays a strategic role in the regulation of their differentiation. Here, we evaluated the M2 muscarinic receptor activation in controlling hdASC proliferation and stabilization of the hdASC phenotype. In accordance with our data in rats, M2 stimulation results in a reversible decrease of cell growth and migration in hdASCs, negatively modulates proliferation markers and upregulates differentiation markers. Remarkably, hdASC differentiation can be stabilized by M2 receptor activation in the absence of differentiation media maintaining a spindle-shaped morphology and SC-like marker expression. These results show that the M2 receptor enhances the hdASC phenotype, maintaining the expression of key glial markers and supporting their pro-regenerative properties.

## Introduction

Schwann cells (SCs) are an attractive cell therapy tool for several peripheral nerve pathologies, including neuropathies and spinal cord and peripheral nerve injury (PNI) [1–4]. However, transplanting autologous SCs presents several limitations: isolation requires harvest of a healthy peripheral nerve, thereby conferring a permanent sensory deficit; moreover, in vitro SC proliferation is very slow, greatly limiting effectiveness as a cell therapy candidate for acute nerve injuries.

Adult mesenchymal stem cells (MSCs) possess high proliferative rates and multipotency; they can be easily isolated from bone marrow and fat tissue [5, 6]. Differentiation of MSCs towards a SC-like phenotype was firstly performed by Dezawa in rat bone marrow MSCs with exposure to a combination of growth factors: forskolin (fsk), glial growth factor 2 (GGF-2), basic fibroblast growth factor (bFGF), and platelet-derived growth factor (PDGF) [6]. This protocol has been subsequently employed in rat and human adipose-derived stem cells (ASCs) towards a SC-like phenotype (dASCs) [7–9], which after 18 days of treatment, express specific glial markers as p75NTR, s100β and GFAP [10–14]. In models of peripheral nerve injury (PNI), rat dASCs promote neurite outgrowth in vitro, reduce neuronal cell death [15] and enhance peripheral nerve regeneration in vivo [16, 17]. These effects are likely to be mediated at least in part by dASCs’ ability to secrete growth factors (i.e., BDNF, NGF) [18, 19]. Whilst human dASCs (hdASCs) share a similar neurochemical profile and excellent potential in PNI therapy, these cells lose their phenotype following the withdrawal of differentiation media [8]. This represents a strong limitation for their potential use in clinical therapy. In spite of that, improving and stabilizing dASC phenotype following the withdrawal of differentiation media could be an indispensable requirement for their clinical application in nerve regeneration therapy.

Multiple molecules have been suggested as potential pharmacological modulators of dASC and SC physiology. Neurotransmitters, such as γ-aminobutyric acid (GABA), adenosine triphosphate (ATP), glutamate, and acetylcholine (ACh), and their receptors have been proposed as potential therapeutic targets for nerve repair [20, 21].

Over the last two decades, ACh has emerged as a critical mediator to multiple neuronal/glial physiological events, glial differentiation and myelination [20, 21]. Our group have demonstrated that ACh, via M2 muscarinic receptor (mAChR), confers significant functional effects on both rat and human SCs, reducing proliferation, promoting the pro-myelinating phenotype and upregulating the expression of myelin proteins, MBP and P0 [21–27]. Furthermore, mAChR stimulation in rat dASCs causes an enhancement of the spindle-shaped morphology, a reversible inhibition of cell growth and migration alongside a downregulation of the pro-apoptotic isoform of NGF (pro-NGF-B) [19, 28–30].

Herein we evaluate the functional effects of M2 mAChR activation in human dASC proliferation and migration, addressing the possible role of M2 mAChR in the stabilization of their differentiation. We demonstrate that hdASCs express muscarinic receptors and that the selective stimulation of M2 mAChR leads to a reversible decrease of cell growth, modulates c-Jun and Egr2 expression, master regulators of SC phenotype, and significantly reduces hdASC migration. Additionally, the M2 receptor contributes to the stability of the hdASC phenotype following the withdrawal of differentiation media, which subsequently supports their functional role in promoting neurite outgrowth, both in embryonic and adult dorsal ganglia root neurons (DRGs).

## Results

Stimulation of M2 receptors in both rat and human SCs negatively regulates cell proliferation and improves the promyelinating phenotype [22–24]. In rat ASCs [29] and dASCs [30] M2 stimulation inhibits proliferation; however, their role in human dASC physiology remains unexplored. Recognising the role of M2 receptors in the regulation of SC differentiation, we hypothesized that they will control human dASC phenotype towards a more stable SC-like phenotype. Therefore, we explored dASC physiology and differentiation in the presence of the M2 mAChR agonist Arecaidine Propargyl Ester (APE).

### Human dASCs express muscarinic receptors

**Fig. 1:**
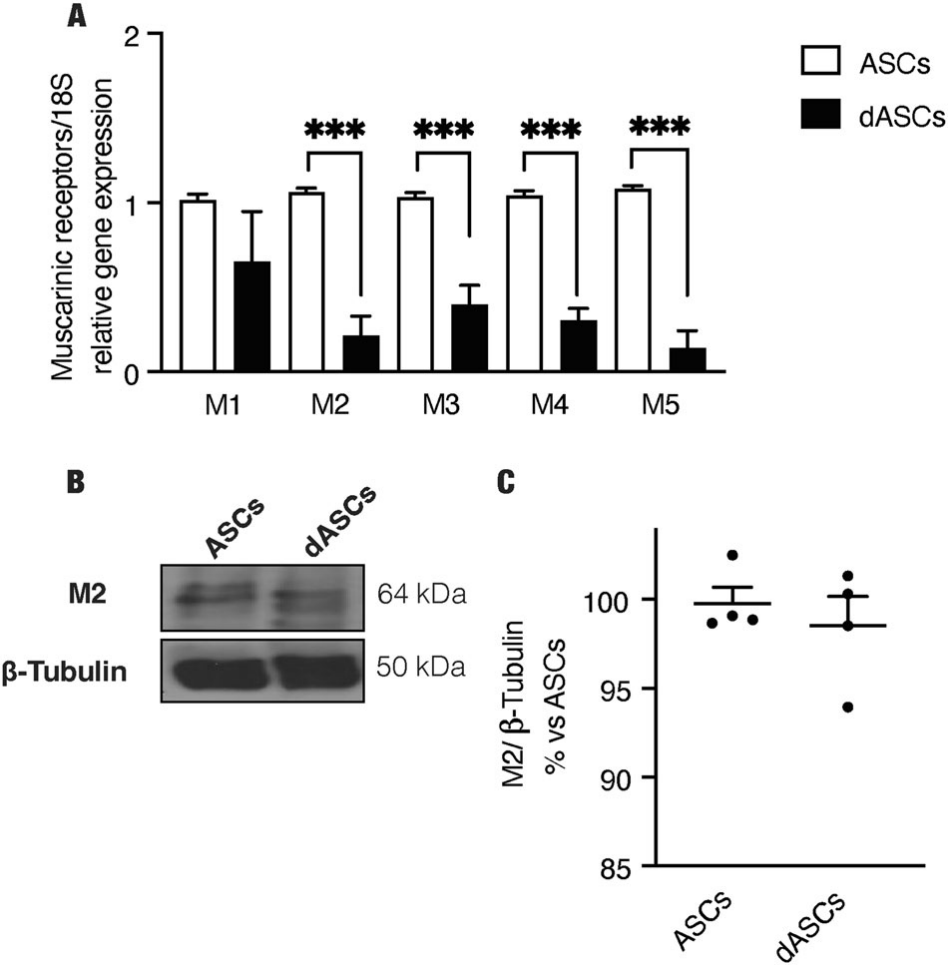
Muscarinic receptors are expressed by hdASC. **A** qRT-PCR expression of muscarinic receptor subtypes. After dASCs differentiation, a significant downregulation of all muscarinic subtypes was observed. The data are the average ± SEM of four independent experiments performed in triplicate [*P* < 0.001(***); *n* = 4]. **B** Representative western blotting of M2 muscarinic receptors protein expression. β-tubulin was used as a protein reference. Densitometric analysis of M2 protein levels showed no significant difference between undifferentiated and differentiated ASCs from the same patient (**C**, *n* = 4).

Gene expression data showed that, after differentiation, a downregulation of all acetylcholine muscarinic subtype (M1-M5) transcripts was observed in human dASCs (Fig. 1A). Protein expression demonstrated a broadly similar M2 receptor profile in human ASCs and dASCs of the same patient (Fig. 1B, C). To evaluate the different receptor expressions during differentiation, human ASCs and subsequently dASCs from the same patient are matched in each experiment.

### M2 receptor stimulation inhibits cell growth and migration, promoting dASC differentiation

**Fig. 2:**
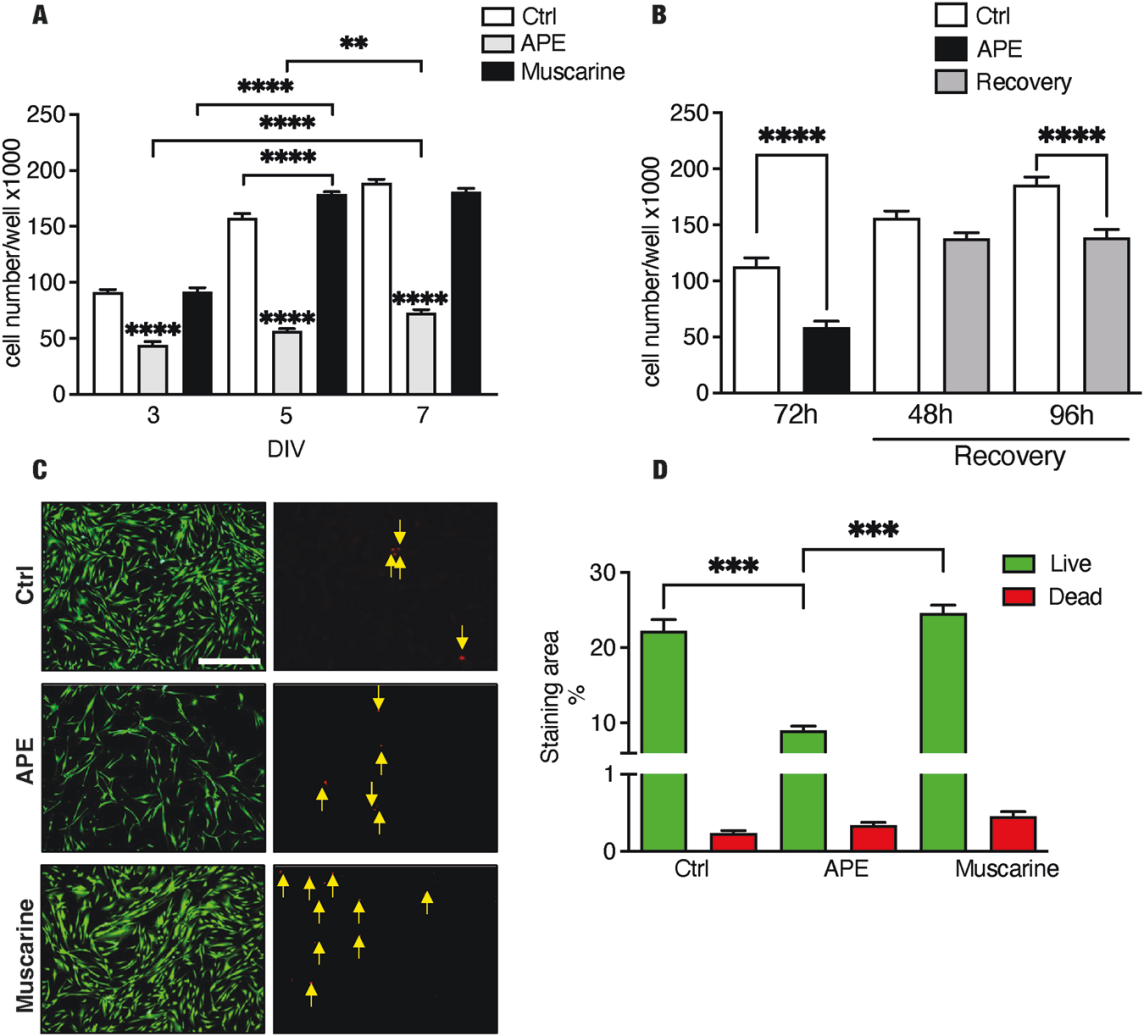
M2 receptor selective activation inhibits cell growth in hdASCs. **A** M2 activation decreased cell growth after 72 h of treatment up to 7 days [*P* < 0.01(**), and *P* < 0.0001(****); *n* = 4]. Although a significant difference between muscarine treatment and untreated cells after 5 days of exposure [*P* < 0.0001(****); *n* = 4], muscarine treatment did not affect cell growth and cell number was comparable to control after 7 days. **B** After APE withdrawal from the culture media, a significant recovery of cell growth was observed. Recovery analysis suggests that the inhibition of M2-mediated cell growth is reversible [*P* < 0.0001(****); *n* = 4]. **C**, **D** Live-dead assay: APE and muscarine treatments had any effects on cell survival [live cells = green; dead cells = red; scale bar = 400 µm; *P* < 0.001(***); *n* = 4] while the reduction of live cells confirms a decrease of cell proliferation-APE induced.

**Fig. 3:**
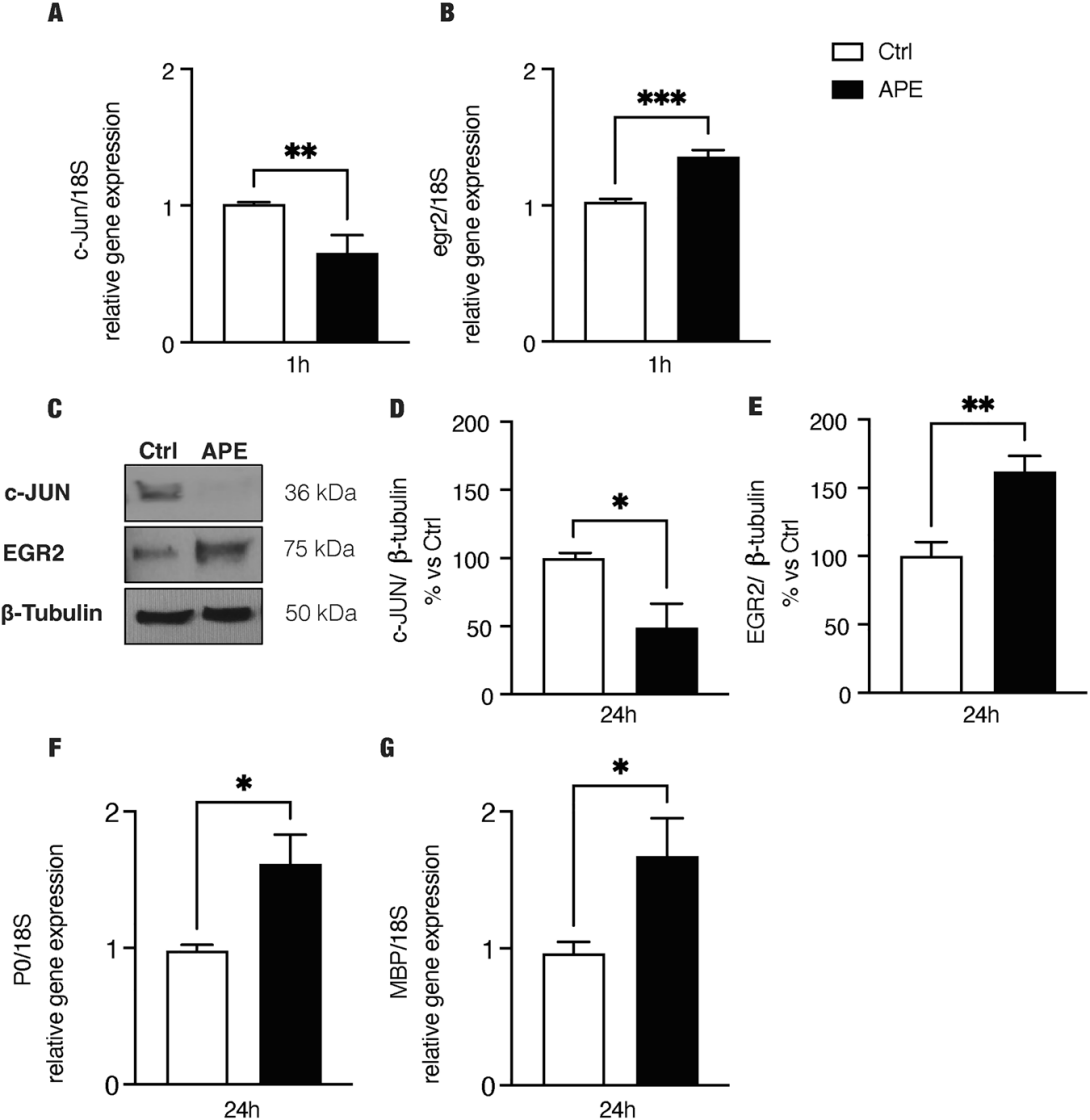
M2 activation downregulates c-jun and upregulates egr2 and myelin proteins. M2 selective activation downregulated in hdASCs the proto-oncogene c-jun transcript expression (**A**; 1 h of treatment, fold-change = 0.655 ± 0.129, **P = 0.0096, *n* = 4) and protein level (**C**, **D**; % vs. Ctrl: 48.85 ± 17.80, *P < 0.05, *n* = 4), whereas upregulates egr-2 expression both as transcript and as protein [(**B**; 1 h of treatment, fold-change = 1.357 ± 0.053, ***P = 0.0004, *n* = 4); **C**, **E**; 24 h of treatment, % vs. Ctrl: 162.0 ± 11.6, **P = 0.0067, *n* = 4)]. **F**, **G** Analysis by qRT-PCR showed an upregulation of the transcript levels of myelin proteins P0 (F; fold-change: 1.616 ± 0.213, *P < 0.05, *n* = 4) and myelin basic protein, MBP (**G**, fold-change: 1.672 ± 0.2794; *P < 0.05, *n* = 4).

**Fig. 4:**
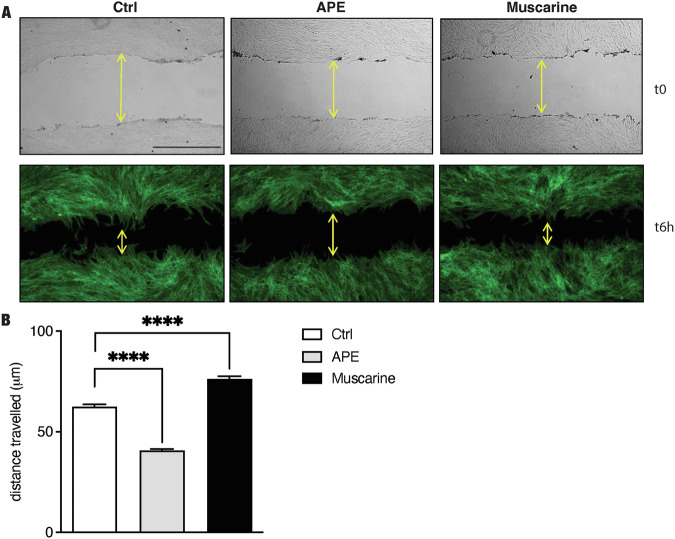
M2 selective activation inhibits cell migration. **A** Pictures were taken at time 0 and after 6 h from the scratch. **B** The graph reported the distance traveled (µm) in 6 h; APE treatment significantly decreased cell migration (distance traveled: 40.7 ± 0.82 µm, ****P < 0.0001, *n* = 4); while the non-selective muscarinic receptors agonist, muscarine, increased cell migration (distance traveled: 76.29 ± 1.35 µm, ****P < 0.0001, *n* = 4). Scale bar = 400 µm.

hdASCs treated with APE and muscarine were analyzed by MTS assay up to 7 days in vitro (DIV). M2 receptor activation using 100 μM of APE exposure inhibited cell growth from 3 days of treatment (Fig. 2A) and persisted for at least 7 days. Conversely, activation of all muscarinic subtypes via muscarine treatment increased cell proliferation at 5 days of exposure, potentially indicating a differential effect of the muscarinic subtypes in the control of hdASC cell proliferation (Fig. 2A). The cell growth inhibition is reversible and not influenced by cell death. Recovery assay showed that after M2 agonist withdrawal after 3 days of treatment, hdASCs were able to recover their proliferative rate (Fig. 2B). The analysis of cell viability performed after 72 h of treatments showed that any pharmacological treatment induced significant toxicity, therefore the reduced cell growth caused by APE treatment was not dependent on cell death (Fig. 2C, D). Interestingly, similar to that observed in rat SCs [23], APE treatment significantly decreased c-jun transcript after 1 h (Fig. 3A) and protein level after 24 h of treatment (Fig. 3C, D), whereas Egr2 was significantly upregulated after APE exposure, both as transcript (Fig. 3B) and as protein (Fig. 3C, E). Egr2 upregulation was accompanied by the increase of transcript levels for the myelin protein P0 (Fig. 3F) and myelin basic protein (MBP) after 24 h of treatment (Fig. 3G). Moreover, as demonstrated for rat SCs [25] and dASCs [30], cell migration (Fig. 4A, B) was impaired after APE treatment, whereas muscarine treatment was able to significantly trigger cell migration compared to untreated cells (Fig. 4A, B).

### M2 selective stimulation stabilizes dASC phenotype, maintaining S100β, EGR2 and neurotrophic factors expression

**Fig. 5:**
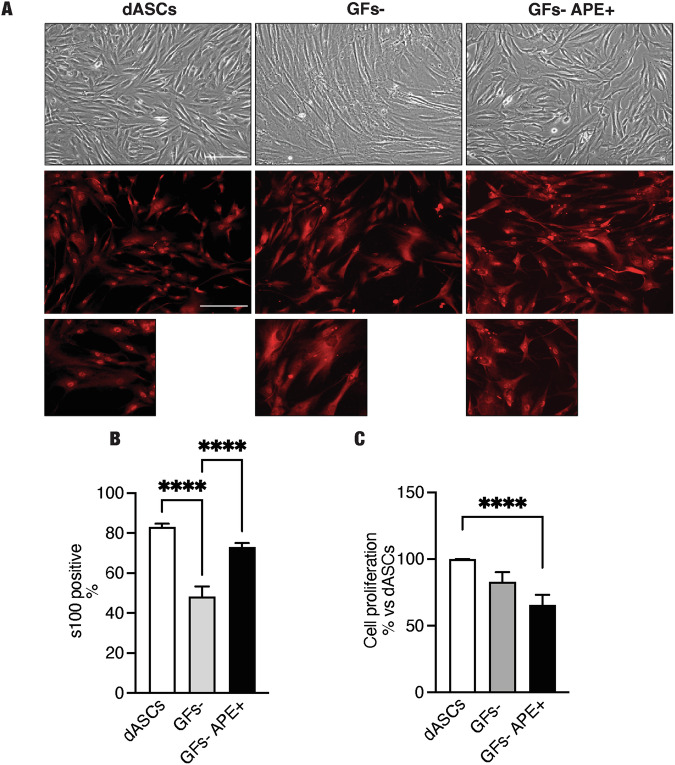
Stabilization of human dASC phenotype after growth factors withdrawal. **A** After growth factors withdrawal, as showed in the light microscope and S100 β immunostaining, cells showed a fibroblast-like morphology, whereas when they were treated with only M2 agonist APE, morphology was similar to dASCs (scale bar: 100 µm). **B** In differentiation media condition, the percentage of S100β positive cells was 83.12 ± 1.59% (*n* = 7). After growth factors withdrawal, a significant reduction of the percentage of S100β positive cells was observed [GFs-, S100β positive cells: 48.30 ± 4.95%, ****P < 0.0001, *n* = 7], whereas, when treated with APE, the percentage was not significantly modified compared with dASCs, used as control (GFs-APE, S100β positive cells: 73.05 ± 1.97%). Scale bar = 200 µm. **C** MTS assay showed a reduction of cell growth after growth factors withdrawal that it was more evident after APE treatment [percentage of cell growth: 65.63% ± 7.521, *P* = 0.0031 (*n* = 7)].

**Fig. 6:**
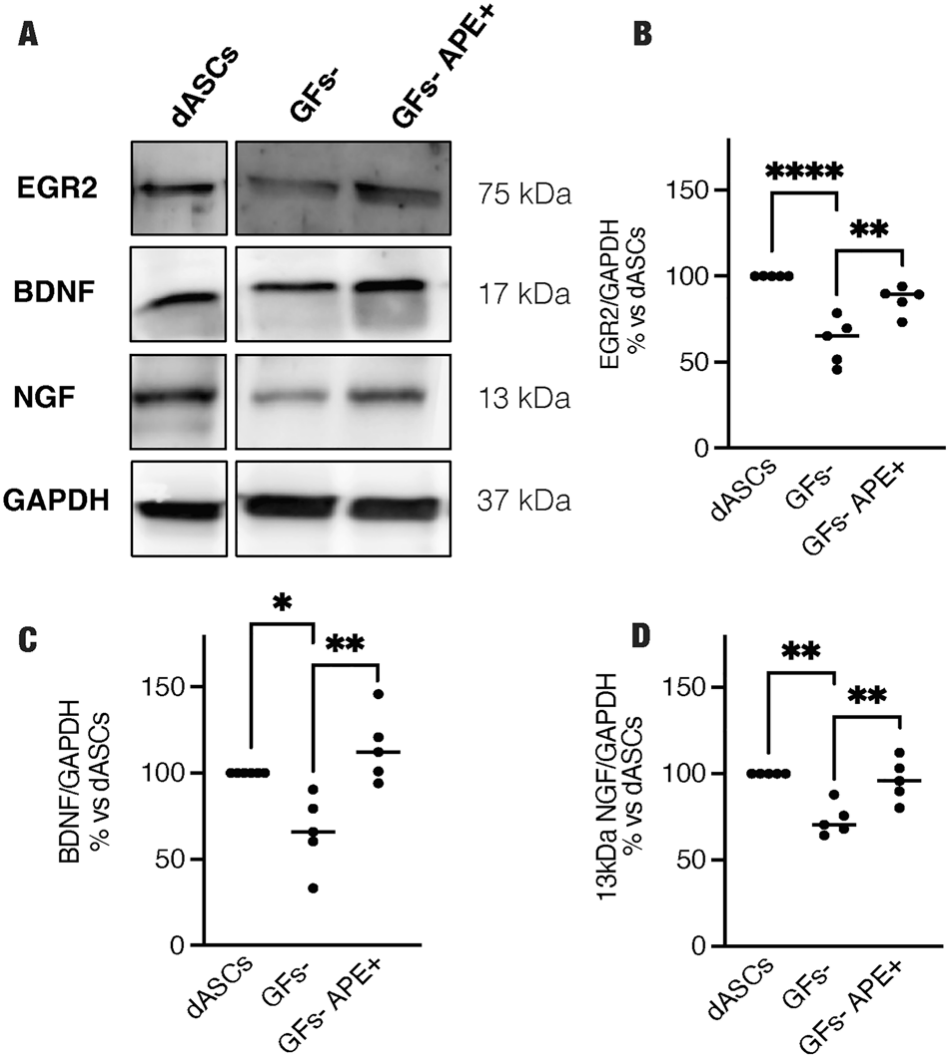
M2 receptor activation maintains Egr2, NGF and BDNF protein expression. **A** Western blot analysis for different SC markers. **A**, **B** Egr2 was significantly downregulated after growth factor withdrawal (GFs-: 62.11 ± 6.0% vs dASCs, ****P < 0.0001, *n* = 5) but cells treated with APE after growth factors withdrawal (72 h) showed similar dASC protein levels of Egr2 (**B**, GFs-APE + : 86.23 ± 3.56% vs dASCs; GFs- vs GFs-APE + : **P < 0.001, *n* = 5). **A**, **C**, **D** BDNF and NGF 13 kDa protein expression were downregulated after growth factor withdrawal (**C**, BDNF GFs-: 65.76 ± 9.734% vs dASCs, *P < 0.05; BDNF GFs- vs GFs-APE + : **P < 0.001, *n* = 5); (**D** NGF, GFs-: 73.26 ± 4.08% vs. dASCs, **P < 0.001; NGF GFs- vs GFs-APE + : **P < 0.001, *n* = 5), whereas APE exposure maintained a similar protein level compared with dASCs. For quantification of western blot analyses, protein levels expression was normalized for the reference protein GAPDH, used as a loading control, and expressed as percentage vs dASCs ± SEM (*n* = 5).

One of the limits of the potential use of dASCs in regenerative medicine is the evidence that the withdrawal of differentiation media causes a reversion of the dASC to ASC phenotype [8]. As shown in Fig. 5A, after growth factor withdrawal (GFs-), hdASCs quickly reverted to fibroblast-like morphology, typical of ASCs [8, 9]. Otherwise, cells treated with APE after growth factor withdrawal (GFs- APE + ) preserved a spindle-shaped morphology, characteristic of ASC differentiated towards SCs phenotype (dASC) (Fig. 5A, GFs- APE + ). The results were also confirmed by the immunostaining for S100β, one of the key glial markers expressed by SCs [4]. In order to evaluate the number of SC-like cells/field, the percentage of S100β positive cells versus DAPI staining in different culture conditions was measured (Fig. 5A, B). After the withdrawal of differentiation media, a significant reduction of S100β positive cells was observed (Fig. 5B), whereas APE treatment was able to maintain the percentage of the S100β positive cells comparable to hdASCs maintained in differentiation media (Fig. 5B). Cell proliferation was significantly reduced following the withdrawal of differentiation media compared with hdASCs after 72 h (Fig. 5C) and this reduction was more evident when GFs- cells were treated with APE 100 µM (Fig. 5C), according to the previous results shown in Fig. 2A. This result was corroborated by western blot analyses, where key glial markers and neurotrophic factors were analyzed (Fig. 6A). Egr2 was significantly downregulated after withdrawal of differentiation media (Fig. 6A, B), instead APE treatment in absence of the growth factors media maintained Egr2 protein level expression comparable to dASCs (Fig. 6A, B). Similarly, protein levels of neurotrophic factors NGF and BDNF were downregulated when differentiation media was removed (Fig. 6A, C, and D), but in the presence of APE, their protein expressions were maintained at comparable levels of dASC (Fig. 6A, C, and D).

### M2 receptor stimulation promotes dASC regenerative properties

**Fig. 7:**
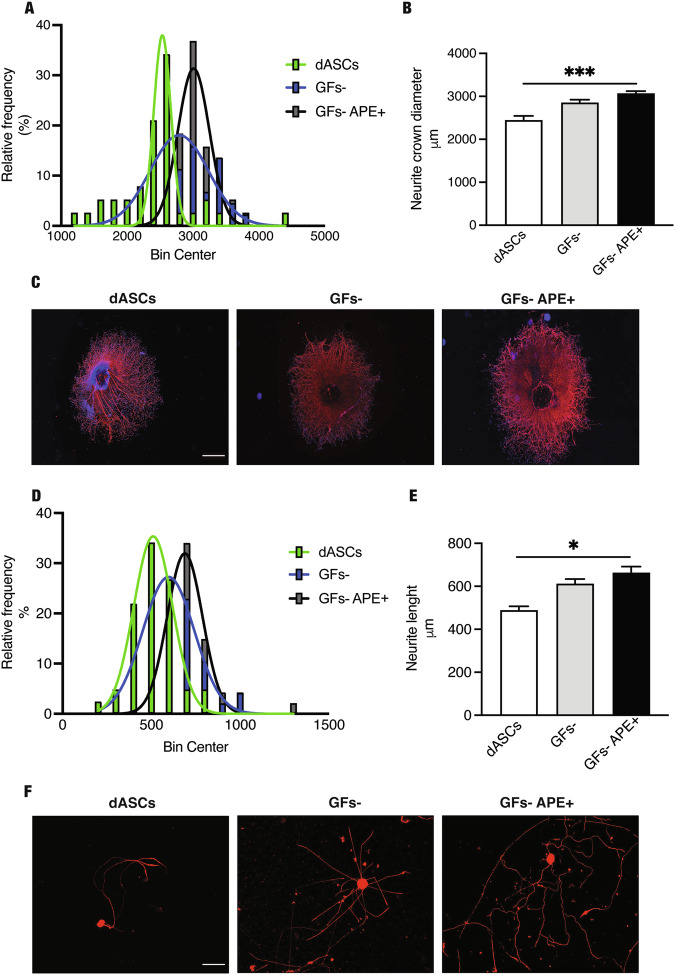
Analysis of neurite length in eDRGs and adult DRGs. eDRGs explants and adult DRGs neurons were treated with hdASC conditioned media derived from 7 different patients [**C**, eDRGs (scale bar = 200 µm) and **F** adult DRGs (scale bar = 100 µm)]. The width of the neurite crown and the neurite length were measured respectively in eDRG explants and in adult DRG neurons. **A**, **D** graphs showed the percentage of a determined measurement of the width of the neurite crown and the neurite length. ANOVA analysis with Bartlett’s test showed a significant increase of the neurite length after conditioned media obtained from hdASC maintained in the presence of the agonist APE both in eDRGs and adult DRGs (**B**, ***P < 0.001; **E**, *P < 0.05).

Considering the ability of M2 receptor to promote rat dASC neurotrophic factor production [19], we evaluated whether hdASCs have positive effects on neurite outgrowth after M2 stimulation. To perform this analysis, eDRGs explants and cultures of dissociated sensory neurons obtained from adult DRGs were treated with conditioned media obtained from hdASC cultures deprived of differentiation media, as described in the methods section. eDRGs treated with the media from hdASCs treated with APE demonstrated a greater neurite crown than those produced in the absence of APE (Fig. 7A, B, C). Moreover, the frequency distribution of the neurite length was included between 2800–3500 µm upon APE treatment, differently from dASCs maintained in absence of APE that presented a frequency distribution within 2400 µm (Fig. 7A). Similarly, media from hdASCs-APE-treated triggered neurite outgrowth also in adult dissociated sensory neurons, as indicated by longer neurites for single neurons (Fig. 7D, E, F). In both conditions, the length of the neurites significantly increased in hdASC media-APE treated compared with the media maintained both in the absence or presence of GFs; the frequency distribution of the neurite length was significantly higher in the presence of APE (700–800 µm) than in dASCs (Fig. 7D; 400–600 µm).

## Discussion

ASCs are multipotent stem cells that can be isolated easily from subcutaneous adipose tissue [31]. This clinically viable approach has led to investigation on their use as SC-like cells, demonstrating their potential therapeutic effects in experimental models of peripheral injury [5, 6, 10, 32–35]. ASCs have a greater proliferation rate than SCs in vitro, and when differentiated from dASCs, their neurotrophic factor expression profile is favorable for nerve regeneration [7, 10, 14, 34, 36].

Seeking to address the unstable phenotype of hdASCs following the withdrawal of differentiation media, new pharmacological targets have been explored. Here we studied the ACh and muscarinic receptor as a promising therapeutic tool for peripheral nerve repair strategies. ACh has well-described functions in neurotransmission; however, alternative roles for ACh in diverse tissues [37–42] as well as its ability to modulate neurite outgrowth [43, 44] have been largely described. Moreover, El-Habta and co-authors [45] clearly demonstrated that rat ASCs express transcripts for *Chat* and *Slc18a3*, the vesicular ACh transporter, and an increase of ChAT protein level is observed in dASCs compared with undifferentiated cells, demonstrating a possible autocrine/paracrine effect of ACh in these cells. We previously investigated the presence and role of ACh muscarinic receptors, and in particular of M2 muscarinic subtype, before in rat and human SCs [22–24] and then in rat ASCs [29] and dASCs [19, 30], demonstrating that the selective stimulation of M2 muscarinic receptor subtype, using the orthosteric agonist APE, negatively modulated cell growth and migration, promoting SC and dASC differentiation and neurotrophic factor production. Although hdASCs express all the transcripts for muscarinic subtypes, their expression was significantly downregulated compared with ASCs but M2 protein level results comparable between undifferentiated and differentiated ASCs, even though a different pattern of glycosylation may be present. Building on our previous results in rat, here we confirm the role of the M2 mAChR also in hdASC physiology demonstrating that M2 agonist APE but not muscarine caused a significant decrease of cell proliferation, similarly to that observed in rats [30]. It is relevant that neither APE nor muscarine treatment has toxic effects on hdASCs as largely validated also in other cell types [22, 29, 30, 46]. As observed in rat ASCs [29], dASCs [30] and SCs [22], the decreased proliferation was reversible; APE withdrawal from the culture medium allowed the rescue of dASC proliferation, contributing to maintaining the well-known plasticity typical of SCs [47]. The dASC phenotype is further supported by the significant downregulation of c-jun [48] and upregulation of egr2 expression, a transcriptional factor specifically required for the transitions into the pro-myelinating stages [49, 50] that is, in fact, followed by an upregulation of P0 and MBP gene expression. These results suggest a differentiating effect of the M2 receptor, also in hdASCs, towards pro-myelinating phenotype, which is further confirmed by a more marked spindle-like morphology of the cells when treated with M2 agonist [51]. Cell migration plays an important role in the therapeutic potential of stem cells; ASCs migrate towards the tissue injury region, responding to high levels of chemokines and growth factors present at the injury site [52]. In vitro, M2 receptor activation can decrease hdASC cell migration, whereas all muscarinic subtype activation promoted it, indicating a positive effect of other subtypes, probably M1 and M3, in the control of cell migration. Altogether these data clearly demonstrate that M2 selective stimulation contributes, in accordance with the previously published rat data, to promote the differentiated phenotype of hdASCs.

Dezawa and co-authors described rat mesenchymal stem cells from bone marrow could be differentiated towards a SC phenotype [6]; then the same differentiation protocol was used in rat and human ASCs [8, 10]. Interestingly the growth factor differentiation media withdrawal caused a rapid reversion to stem cell-like morphology of human dASCs [8, 9]. It is, therefore, established that hdASCs may mimic SC phenotype but the effectiveness of dASC differentiation is not yet convincing and requires further work before their potential translation in regenerative medicine. SCs demonstrate great plasticity [53, 54]; during early development stage, they assume an immature proliferating phenotype, then differentiate towards a mature phenotype (myelinating or non-myelinating cells) and, additionally, after axonal damage they assume a ‘*Repair’* phenotype, able to promote nerve regeneration [51, 55]. In each phase of SC development, different signals contribute to this complicated physiological program and several of them are dependent on autocrine mechanisms produced by SCs themselves and/or by the neighboring axons [21]. We have demonstrated the role played by Acetylcholine and cholinergic receptors in the SC development and plasticity [21, 25, 56], and M2 muscarinic receptor has a strategic role both in rat and in human SC differentiation [23–26]. This key role has been also found in hdASC, where M2 activation enhanced the maintenance of the spindle-shaped morphology of hdASCs and the persistence of the percentage of S100β positive cells, also after 72 h of growth factors withdrawal. Moreover, our findings showed that dASCs deprived of growth factors and treated with APE maintained high protein levels of Egr2 and neurotrophic factors such as BDNF and mature NGF (13 kDa). These growth factors are widely documented as key regulators of SC response to injury [57]. In the context of an injured environment where growth factor expression changes rapidly, ACh may enhance dASC phenotype and contribute to maintaining high levels of neurotrophic factors that stimulate axon growth and nerve regeneration. To confirm this hypothesis, embryonic and adult DRGs neurons were treated with conditional media derived from hdASC cultures, maintained in the absence of growth factors but treated with APE. After 48 h, an increase in neurite length was more evident both in embryonic and adult DRG neurons compared with those cultured in hdASC conditional media in the absence of APE. Our experimental system did not involve direct contact between hdASCs and embryonic or adult DRGs, suggesting that molecules secreted when stimulated by M2 agonist could improve neurite outgrowth.

In conclusion, considering the relevant role of M2 receptor subtype in regulating rat dASC and SC physiology and differentiation, we found that also in hdASCs, M2 receptor promotes SC-like differentiation. Our study demonstrates a novel role of M2 receptor in the maintenance of hdASC phenotype also in the absence of differentiating growth factors, and in promoting neurotrophic factor production. These two aspects are fundamental in the hypothesis of potential hdASC use as neurotrophic support in peripheral nerve regeneration following injury as they may improve or accelerate the axon regeneration.

## Methods

### Human adipose-derived stem cell harvesting and culture

Human subcutaneous abdominal adipose tissue was withdrawn from 7 consenting patients undergoing reconstructive surgery at Wythenshawe Hospital, Manchester University NHS Foundation Trust, UK. All patients were female, healthy and aged 44–64 years provided informed consent. All procedures were approved by the National Research Ethics Committee, UK (NRES 13/SC/0499), and conformed to the World Medical Association Declaration of Helsinki.

ASCs were isolated as previously described, with minor modifications [10]. Briefly, adipose tissue was minced with a sterile razor blade and enzymatically dissociated with 0.15% (*w/v*) collagenase type I (Life Technologies, Paisley, UK) at 37 °C for 60 min. The resulting digested tissue was filtered through a vacuum-assisted 100 µm nylon mesh (Merck Millipore UK, Watford, UK), and, to neutralize the collagenase action, an equal volume of stem cell growth medium was added [α-MEM (Sigma-Aldrich, Poole, UK) supplemented with 10% foetal bovine serum (FBS, LabTech, Uckfield, UK), 2 mM L-glutamine (GE Healthcare UK, Little Chalfont, UK), and 1% (*v/v*) penicillin–streptomycin solution (Sigma-Aldrich, UK)]. Cells were centrifuged at 1200 rpm for 10 min, and the resulting pellet [stromal vascular fraction (SVF)] was resuspended in 1 ml of Red Blood Cell Lysis Buffer (Sigma-Aldrich, UK) for 1 min; then 20 ml of α-MEM was added to arrest cell lysis. The mixture was centrifuged at 900 rpm for 10 min, and the resulting pellet was resuspended in α-MEM and plated in T75 flasks for cell culture.

### Differentiation to a Schwann-like phenotype

Human ASCs were differentiated into dASCs by the use of previously described protocol [10, 12]. Sub-confluent cultures of ASCs, passage 1–2 with ASCs at 30% confluence, were incubated with growth media supplemented with 1 mM β-mercaptoethanol (Sigma-Aldrich, UK) for 24 h. Cells were washed twice with Hank’s Balanced Salt Solution (HBSS, Sigma-Aldrich, UK) and followed by pre-conditioning in 35 ng/ml all-trans-retinoic acid (Sigma-Aldrich, UK) for 72 h. After 3 days, cells were washed twice and transferred to differentiation media. The differentiation medium consists of stem cell growth medium supplemented with 5 ng/ml platelet-derived growth factor (PDGF; PeproTech Ltd., London, UK), 10 ng/ml basic fibroblast growth factor (bFGF; PeproTech Ltd., London, UK), 14 μM forskolin (Fsk; Sigma-Aldrich, UK) and 192 ng/ml glial growth factor-2 (GGF-2, Acorda Therapeutics, Ardsley, NY, USA). Cells were incubated in cell differentiation media for 2 weeks.

### Cell treatment with cholinergic agonist and experimental setup

Human dASCs were incubated in their respective media with M2 selective agonist Arecaidine Propargyl Ester (APE, Sigma-Aldrich, UK) at the final concentration of 100 µM, according to the experimental plan. Pharmacological competition with M2 antagonist methoctramine and silencing of M2 receptors by siRNAs have already largely validated APE selectivity [22, 29, 30, 58, 59]. Moreover, hdASCs were treated with the non-selective muscarinic receptors agonist, muscarine (Sigma-Aldrich, UK), for all muscarinic subtypes activation, at the final concentration of 100 µM.

MTS, Live-Dead Assay, migration and gene and protein expression analysis were performed in four patients, whereas conditional media experiment and protein analysis after growth factor withdrawal in seven different patients; all experiments were performed in technical and experimental triplicate. Media for the growth factor withdrawal experiment were not pooled together but kept separately. For protein analysis, cells were rinsed with ice-cold phosphate buffer solution (PBS) and scraped in RIPA lysis buffer (Sigma-Aldrich, UK) containing a cocktail of protease and phosphatase inhibitors (Thermo Fisher Scientific, Loughborough, UK) and the whole cell lysates were used for western blot analysis (see Protein Extraction and Western blot section). For qPCR analysis, cells were scraped with RNA protect cell reagent (Qiagen, Manchester, UK) and then processed as described in ‘Quantitative real-time polymerase chain reaction (qRT-PCR)’.

### Cell proliferation assessment

Human dASCs were plated in 24-well plates (Corning Life Sciences, USA) at a density of 10 × 10^3^ per well in triplicate and treated the day after, as mentioned in ‘Cell treatment with cholinergic agonist and experimental setup’ section. Cell media were changed after 72 h, and treatments were replaced.

At day 3, 5 and 7 after treatment, the medium was aspirated, and cells were incubated in 20% (*v/v*) CellTiter 96 AQueous One Solution Cell Proliferation Assay (Promega, Southampton, UK), diluted in phenol-free DMEM (Sigma-Aldrich, UK). Following 90 min of incubation at 37 °C in the dark, the absorbance at 490 nm was recorded with an Asys UVM-340 microplate reader/spectrophotometer (Biochrom, Cambridge, UK). After a standard curve was performed, data were expressed as cell number ± standard error of the mean (SEM).

To demonstrate whether APE-induced inhibition of cell growth was reversible, a recovery analysis was set up. Cells were treated for 72 h with APE and then were washed once with HBSS, and fresh complete medium was added. Growth analysis was assessed by MTS assay as mentioned above.

### Live-dead assay

Cell viability was analyzed by using the LIVE/DEAD Viability/Cytotoxicity Kit for mammalian cells (Molecular Probes, Invitrogen, UK). Live cells were identified using Calcein-AM (green fluorescence; *λ* = 498 nm), whereas Eth-D1 (red fluorescence; *λ* = 635 nm) was used to observe dead cells. 50 × 10^3^ cells/well of hdASCs were plated into 12-well plates and treated the day after. Calcein-AM and Eth-D1 were added at the final dilution of 0.5 μl/ml and 2 μl/ml, respectively. Cells were incubated for 30 min at room temperature (RT) and then washed in PBS. Images were taken using a fluorescence inverted microscope (Olympus IX51, Southend-on-Sea, UK) under 4x magnification and processed with ImageJ imaging software (NIH, Bethesda, MD, USA). The result was shown as the percentage of the staining of Calcein-AM and Eth-D1/field.

### Quantitative real-time polymerase chain reaction (qRT-PCR)

For gene expression studies, RNA was extracted using RNeasy Plus Mini Kit (Qiagen, Manchester, UK), according to the manufacturer’s protocol. The RNA concentration was determined by spectrophotometric analysis with a NanoDrop ND-100 (Thermo Fisher Scientific, Waltham, MA, USA), and each sample was reverse-transcribed using RT^2^ First Strand Kit (Qiagen, Manchester, UK), agreeing with the manufacturer’s protocol. Both RNA extraction and cDNA synthesis included DNA elimination steps to ensure the absence of downstream genomic DNA amplification.

qRT-PCR was performed with RT^2^ SYBR Green qPCR Mastermix (Qiagen, Manchester, UK) using Corbett Rotor Gene 6000 real-time cycler (Qiagen, UK). All reactions were carried out in triplicate, and the following protocol used: hot start for 10 min at 95 °C, followed by 45 cycles of 15 s at 95 °C, annealing for 30 s at 55 °C and extension for 30 s at 72 °C. To verify the specificity of the reactions, a melting curve was obtained with the following protocol: 95 °C for 1 min, 65 °C for 2 min, and a gradual temperature increase from 65 °C to 95 °C (2 °C/min). All the primer sequences are listed in Table 1. Data were normalized for the housekeeping gene 18S, and the ΔΔCt method was used to determine the fold changes in the gene expression compared with control.

**Table 1:** List of primers.

Gene	Forward	Reverse
RN18S1	ATCGGGGATTGCAATTATTC	CTCACTAAACCATCCAATCG
MBP	GGAAACCACGCAGGCAAACGAGA	GAAAAGAGGCGGATCAAGTGGGG
P0	ACCTCTCAGGTCACGCTGTA	CAGCAGTACCGAACCACGTA
M1	AGAGAGACCCTGCCAACTTT	CTCCTGACTTCCTGCCTAAA
M2	CCAAGACCCCGTTTCTCCAAG	CCTTCTCCTCTCCCTGAACAC
M3	CGCTCCAACAGGAGGAAGTA	GGAGTTGAGGATGGTGCTGT
M4	AATGAAGCAGAGCGTCAAGAA	TCATTGGAAGTGTCCTTATCA
M5	CCTGGCTGATCTCCTTCATC	GTCCTTGGTTCGCTTCTCTG
EGR2	AACGGAGTGGCCGGAGAT	ATGGGAGATCCAACGACCTCTT
c-jun	GCATGAGGAAACGCATCGCTGCCTCCAAGT	GCGACCAAGTCCTTCCCACTCGTGCACACT

### Protein extraction and Western blot

Whole-cell lysates were obtained using RIPA Buffer (Sigma-Aldrich, UK) supplemented with protease and phosphatase inhibitors (Thermo Fisher Scientific, Loughborough, UK). Lysates were incubated for 30 min on ice and later centrifuged for 20 min at 14000 rpm at 4 °C. Protein concentration was determined using Pierce^TM^ BCA Protein Assay Kit (Thermo Fisher Scientific, Waltham, MA, USA), according to the manufacturer’s protocol.

Sample buffer (6×) was added to protein lysates and heated for 5 min at 100 °C. Thirty μg of each sample was loaded onto a 10 or 15% SDS (Sodium dodecyl sulfate) polyacrylamide gel and run at 120 V using Tris-glycine running buffer [25 mM Tris, 190 mM glycine, 0.08% (*w/v*) SDS]. Resolved proteins were transferred for 60 or 90 min (according to protein molecular weight) onto nitrocellulose blotting membranes (GE Healthcare Life Science, Amersham, Germany) at 80 V or 30 V, according to protein molecular weight, respectively, in a transfer buffer (25 mM Tris-base; 192 nM glycine, 20% (*v/v*) methanol). In order to confirm successful protein transfer, membranes were stained with Ponceau red (Sigma-Aldrich, Poole, UK), before being blocked for 1 h in a Tris-buffer saline (TBS)-Tween Solution (10 mM Tris pH 7.5, 100 mM NaCl, 0.1% (*v/v*) Tween) containing 5% (*w/v*) of non-fat dry milk (blocking buffer). Membranes were incubated with the primary antibody, diluted in the blocking buffer, overnight at 4 °C.

After overnight incubation, membranes were washed 5 times with TBS-Tween buffer and thus incubated for 1 h at RT with anti-rabbit or anti-mouse horseradish peroxidase (HRP) secondary antibody (1:2000 and 1:1000, respectively; Cell Signaling, Hitchin, UK) for chemiluminescence detection. Primary antibodies were reported in Table 2. To determine housekeeping protein (GAPDH or β−tubulin), membranes were stripped with a glycine solution (100 mM, pH 2.9; Sigma-Aldrich, UK) for 15 min at RT and, after washes, re-blocked again, as previously reported, prior to further blotting.

**Table 2:** List of antibodies.

Primary antibody (catalog, manufacture, dilution)	Secondary antibody (catalog, manufacture, dilution)
mouse anti-**M2** Abcam ab2805 1:800	**HRP-linked anti-mouse IgG** Sigma 1:1000
rabbit anti-**EGR2** Proteintech 13491-1-AP 1:500	**HRP-linked anti-rabbit IgG** Sigma 1:2000
rabbit anti-**c-jun** Abcam ab31419 1:1000	**HRP-linked anti-rabbit IgG** Sigma 1:2000
rabbit anti-**NGF** Alomone AN-240 1:400	**HRP-linked anti-rabbit IgG** Sigma 1:2000
rabbit anti-**BDNF** Alomone ANT-010 1:200	**HRP-linked anti-rabbit IgG** Sigma 1:2000
rabbit anti-**GAPDH** Proteintech 10494-1AP 1:5000	**HRP-linked anti-rabbit IgG** Sigma 1:2000
mouse anti-**β-actin** Sigma-Aldrich A5441 1:5000	**HRP-linked anti-mouse IgG** Sigma 1:1000

Membranes were exposed to SuperSignal West Pico Chemiluminescent Substrate (Thermo Fisher Scientific, Waltham, MA, USA) for signal detection.

The optical density (OD) of each protein band was analyzed with ImageJ imaging software (Version 1.52a, National Institutes of Health NIH, United States) and normalized against the OD of the protein reference band.

### Immunocytochemistry

Cells from each experimental group were plated on 13 mm glass coverslips (Avantor VWR international, UK) at a density of 5 × 10^3^ cells/well. For the experiment with DRGs, adult and embryonic DRGs were also plated on 13 mm glass pre-coated coverslips (Avantor VWR International, UK). According to the experimental plan, cells/DRGs were fixed for 20 min in 4% (*w/v*) paraformaldehyde (PFA, Sigma-Aldrich, UK) and washed in phosphate buffer solution (PBS, Sigma-Aldrich, UK). For permeabilization, cells/DRGs were incubated with Triton-X-100 0.2% for 30 min at RT, then were washed twice with PBS and blocked with block solution (PBS 0.1% Triton-X-100) containing, according to secondary antibody, 10% normal donkey serum (NDS) or normal goat serum (NGS) (Sigma-Aldrich, Poole, UK) for 1 h at RT. Cells/DRGs were incubated with primary antibody [S100 (rabbit polyclonal 1:500; Dako, Ely, UK); β-III tubulin (mouse monoclonal, 1:500, T8660, Sigma-Aldrich, UK)], in 0.1% Triton-X-100, 0.1% (*w/v*) BSA, 0.1% (*w/v*) Sodium Azide in PBS at 4 °C overnight. The day after, cells/DRGs were washed with PBS three times for 10 min and they were incubated with secondary antibody [Donkey anti-rabbit IgG (H + L) Highly Cross-Adsorbed Secondary Antibody, Alexa Fluor 568, (Life Technologies, UK); Goat anti-Mouse IgG (H + L) Highly Cross-Adsorbed Secondary Antibody, Alexa Fluor 568, (Life Technologies, UK)] in 0.1% Triton-X-100, 0.1% (*w/v*) BSA, 0.1% (*w/v*) Sodium Azide in PBS for 1 h at RT. Again, cells were washed 3 times with PBS and then mounted with Vectashield mounting medium for fluorescence containing 4’-6’-diamidino-2-phenylindole for nuclear staining (H1200, Vector Lab, DBA, Milan, Italy). Images were taken using a fluorescence microscope (Olympus BX60 wide-field microscope, Southend-on-Sea, United Kingdom) and processed with ImageJ imaging software (v1.52a, National Institutes of Health, NIH, Bethesda, MD, USA).

### Wound healing assay

A wound healing assay was performed as previously described [25, 29, 30]. Briefly, wound was produced with p200 tip and, 50 ng/ml Mitomycin C (Sigma-Aldrich, UK) was added 3 h before to perform the scratch to arrest cell proliferation. For each image, three measurements of the space between two fronts were taken for both time points [time 0 (t0) and time 6 h (6 h)], and the average of the three measurements was calculated. The two values were subtracted (t0–t6h), obtaining the covered space by the cells in the experimental time chosen. Analyses will be performed blind with coded samples.

After wound healing experiment (6 h experiment), cells were fixed for 20 min in 4% (*w/v*) paraformaldehyde (PFA, Sigma-Aldrich, UK), washed in phosphate buffer solution (PBS, Sigma-Aldrich, UK) and stained with phalloidin. Cells were previously permeabilized for 15 min with 0.2% (*v/v*) Triton-X in PBS (Sigma-Aldrich, UK) and stained for 20 min at RT with Alexa 488-conjugated phalloidin (1:40, Life Technologies, UK) diluted in 1% (*w/v*) BSA in PBS. Images were taken using a fluorescence microscope (Olympus IX51 inverted microscope, Southend-on-Sea, UK) and processed with ImageJ imaging software (v1.52a; NIH, Bethesda, MD, USA).

### Maintenance of human dASC phenotype

The effect of M2 muscarinic receptor subtypes on dASC phenotype stability following the withdrawal of the differentiation medium was evaluated after plating 1 × 10^5^ dASCs from each patient in six-well plates in experimental triplicate for each experimental condition, in the growth medium supplemented with growth factors [5 ng/ml PDGF (PeproTech Ltd., London, UK), 10 ng/ml bFGF (PeproTech Ltd., London, UK), 14 μM forskolin (Fsk; Sigma-Aldrich, UK) and 192 ng/ml GGF-2 (Acorda Therapeutics, Ardsley, NY, USA)]. When cells were sub-confluent, medium was aspirated, and cells were washed with Hank’s Balanced Salt Solution (HBSS, Sigma-Aldrich, UK) to remove any residual of growth factors. Cells were then treated either with differentiation medium [dASCs maintained in differentiation medium, i.e. GFs + , dASCs] or with stem cell medium depleted of all growth factors and fsk [dASCs deprived of differentiation medium, i.e., GFs-] and with stem cell medium depleted of all growth factors and fsk plus APE 100 µM treatment [dASCs deprived of differentiation medium plus APE, i.e. GFs- APE + ] for 72 h. At the end of the incubation, supernatants were collected and snap-frozen for DRGs treatment. For the protein expression analyses, cells were washed with ice-cold PBS, collected in RIPA Buffer (Sigma-Aldrich, UK) supplemented with protease and phosphatase inhibitors (Thermo Fisher Scientific, Loughborough, UK) and frozen until further analysis by western blot. For S100β positive cells analyses (polyclonal anti-S100β, Dako, Denmark) 5 × 10^3^ dASCs from each patient were seeded in 13 mm coverslips (Avantor VWR International, UK), and, after the growth factor withdrawal protocol, cells were fixed with PFA and processed as previously described (see ‘Immunocytochemistry’ section).

### Embryonic and adult DRG preparation and conditional media treatments

Embryos were immersed into Leibovitz’s L-15 medium (L-15, Gibco, UK 11415-064). Individual embryos were transferred into a 60 mm dish with a thin layer of L-15 to isolate single embryonic DRGs (eDRG). Coverslips were treated with poly-D-Lysine (Sigma-Aldrich, UK) for 30 min at RT and then with collagen I (Thermo Fisher Scientific, Loughborough, UK) for 2 h at 37 °C. Before plating, coverslips were washed three times with F12 (Sigma-Aldrich, UK). Single eDRG was deposited onto a pre-coated coverslip. DRGs were plated with completed media [minimum essential medium (MEM, Gibco, UK 11090-081) supplemented with 10% FBS (LabTech, Uckfield, UK), 2 g/0.5 L D-Glucose (Sigma-Aldrich, UK), 2 mM L-glutamine (GE Healthcare UK, Little Chalfont, UK) and 50 ng/ml NGF]. The day after, eDRGs were treated with conditional media as described below.

Adult DRGs were dissected in a sterile condition and placed in a 60 mm Petri dish containing F12 medium (Sigma-Aldrich, UK). All roots were cleaned off from connective tissue and placed in a 35 mm petri dish containing F12 (Sigma-Aldrich, UK) supplemented with 1.25% Type IV collagenase (Worthington Biochemical corp, cat. No. LS004188) for 1 h at 37 °C and 5% CO_2_. Type IV collagenase digestion was repeated for 45 min then ganglia were washed gently 3 times with F12 (Sigma-Aldrich, UK). DRGs were incubated with F12 (Sigma-Aldrich, UK) containing 2.5% trypsin (Worthington Biochemical corp, cat. No. LS003703) for 30 min at 37 °C and 5% CO_2_. To neutralize trypsin 1 ml of F12 (Sigma-Aldrich, UK) and 0.5 ml of FBS (LabTech, Uckfield, UK) were added to DRGs and then they were washed 3 times with F12 (Sigma-Aldrich, UK). Ganglia were gently dissociated, and the cell suspension was passed through a sterile 70 μm mesh to remove clumps of undissociated neurons and axon debris. Resulting suspension centrifuged at 900 rpm for 5 min, was layer on top of the 15% BSA, to isolate DRGs neurons from glial cells. Then, the suspension was centrifuged again at 1200 rpm for 10 min. Resulting pellet was resuspended into BS media composed by F12 (Sigma-Aldrich, UK) containing 1x N2 supplement (Thermo Fisher Scientific, Waltham, USA) and 50 ng/ml NGF (Millipore, Massachusetts, USA). Cells were resuspended in an appropriate volume of BS media, then plated in a pre-coated coverslip. Adult DRGs were left for 48 h in BS media before conditional media treatment. For adult DRGs plating, coverslips were treated with poly-D-Lysine (Sigma-Aldrich, UK) for 30 min at RT and then with Laminin (Sigma-Aldrich, UK) for 3 h at 37 °C. hdASCs were cultured for 72 h as the experimental plan described in the previous section. hdASCs media were collected and frozen until DRGs treatment.

Embryonic and adult DRGs were treated for 48 h with conditional media, and then they were fixed with PFA 4% and stained for β-III tubulin (T8660, Sigma-Aldrich, UK) as described in ‘Immunocytochemistry section’. Adult DRGs neurite lengths were measured with ImageJ software (v1.52a; National Institutes of Health, NIH, 469 Bethesda, MD, USA), whereas eDRGs neurite crown diameter was measured with CaseViewer 2.3 (3DHISTECH Ltd., Budapest, Hungary). Analyses will be performed blind with coded samples. Graphs represent the distribution of neurite length in the three experimental conditions.

### Data analysis

Data analyses were performed with GraphPad Prism 8 (Graphpad Software, La Jolla, CA, USA) and presented as the mean ± SEM. Statistical significance for the gene expression was estimated by unpaired two-tailed Student’s *t*-test; for proliferation studies one-way ANOVA analyses with Bonferroni’ or Tukey’s post-hoc tests were used; for migration studies one-way ANOVA analysis with Dunnett post-hoc tests was used. ANOVA analysis with Bartlett’s test was used to analyze eDRGs neurite crown diameter and adult DRGs neurite outgrowth. The densitometric analyses of the qPCR and Western blot were measured by ImageJ software (v1.52a; National Institutes of Health, NIH, 469 Bethesda, MD, USA). The statistical significance was expressed as *P*-values. A value of *P* < 0.05 was considered statistically significant: *P* < 0.05(*), *P* < 0.01(**), and *P* < 0.001(***) *P* < 0.0001(****).

## Supplementary information

Western blot supplemental material

## Data Availability

The data that support the findings of this study are available from the corresponding author upon reasonable request.
