# Supplementary material for: Acetylcholine muscarinic M2 receptor maintains human Schwann-like adipose-derived phenotype in the absence of differentiating medium
Source: Cell Death Discov. 2025 Apr 13;11:170. doi: 10.1038/s41420-025-02404-0 (PMC11994781; doi:10.1038/s41420-025-02404-0)
Supplement: Supplementary file 1 — Western blot supplemental material [file 41420_2025_2404_MOESM1_ESM.pdf]

Figure 1

U= undifferentiated
D= differentiated
SCs= Schwann cells

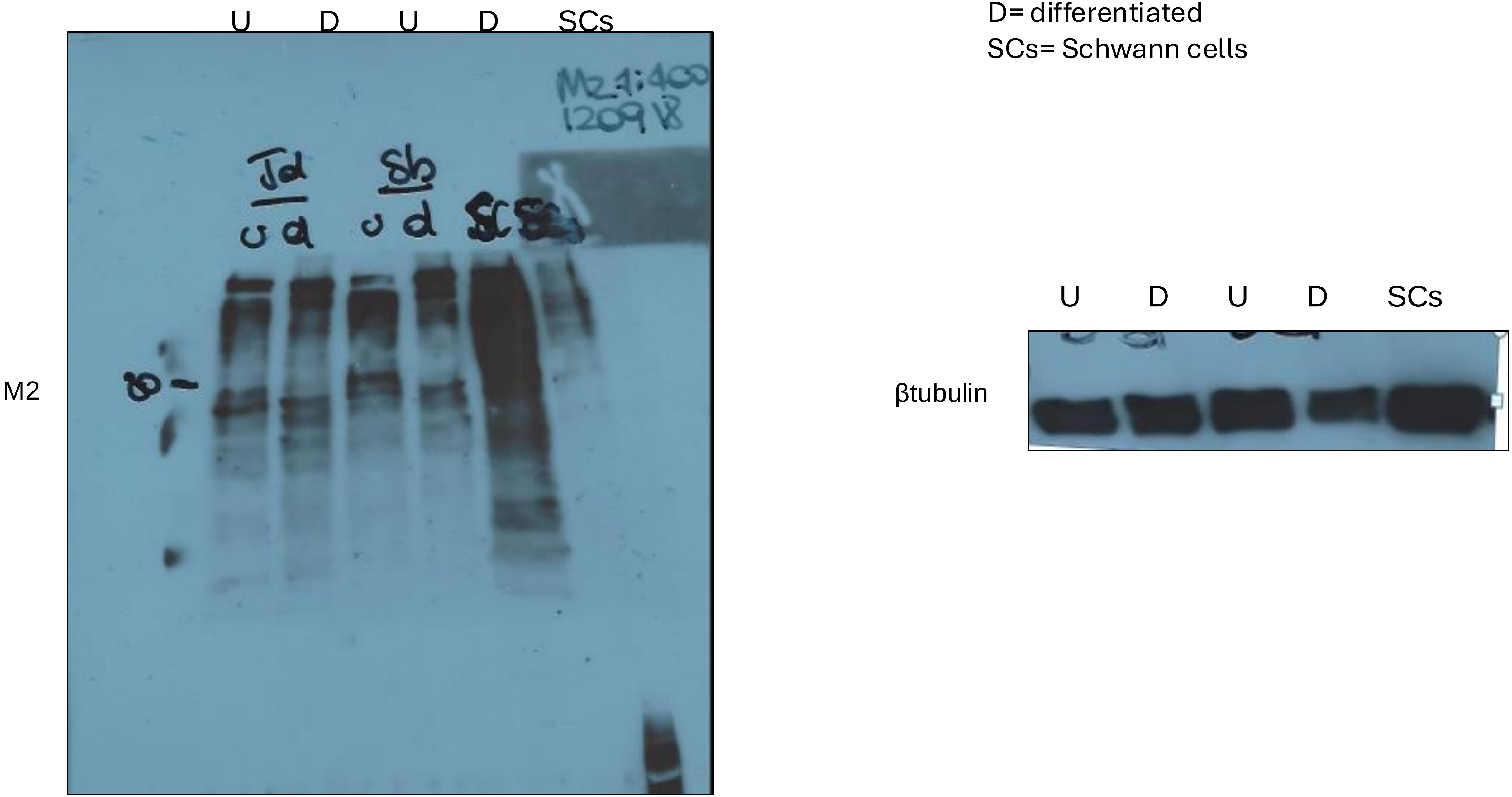

Figure 3

c-Jun

βtubulin

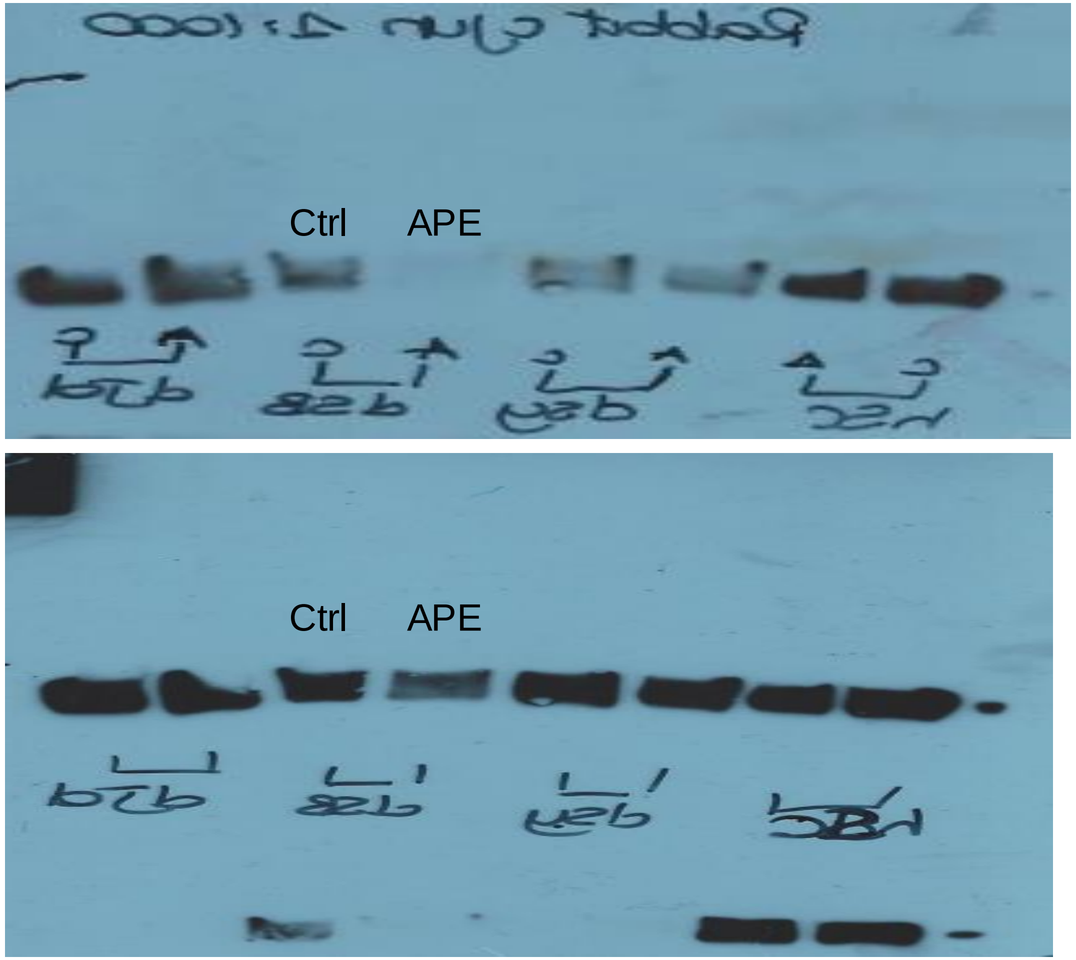

Egr2

βtubulin

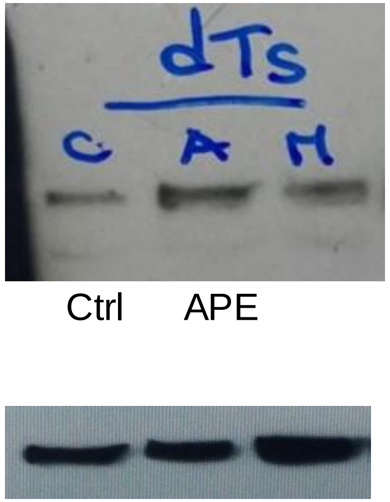

Figure 6

EGR2

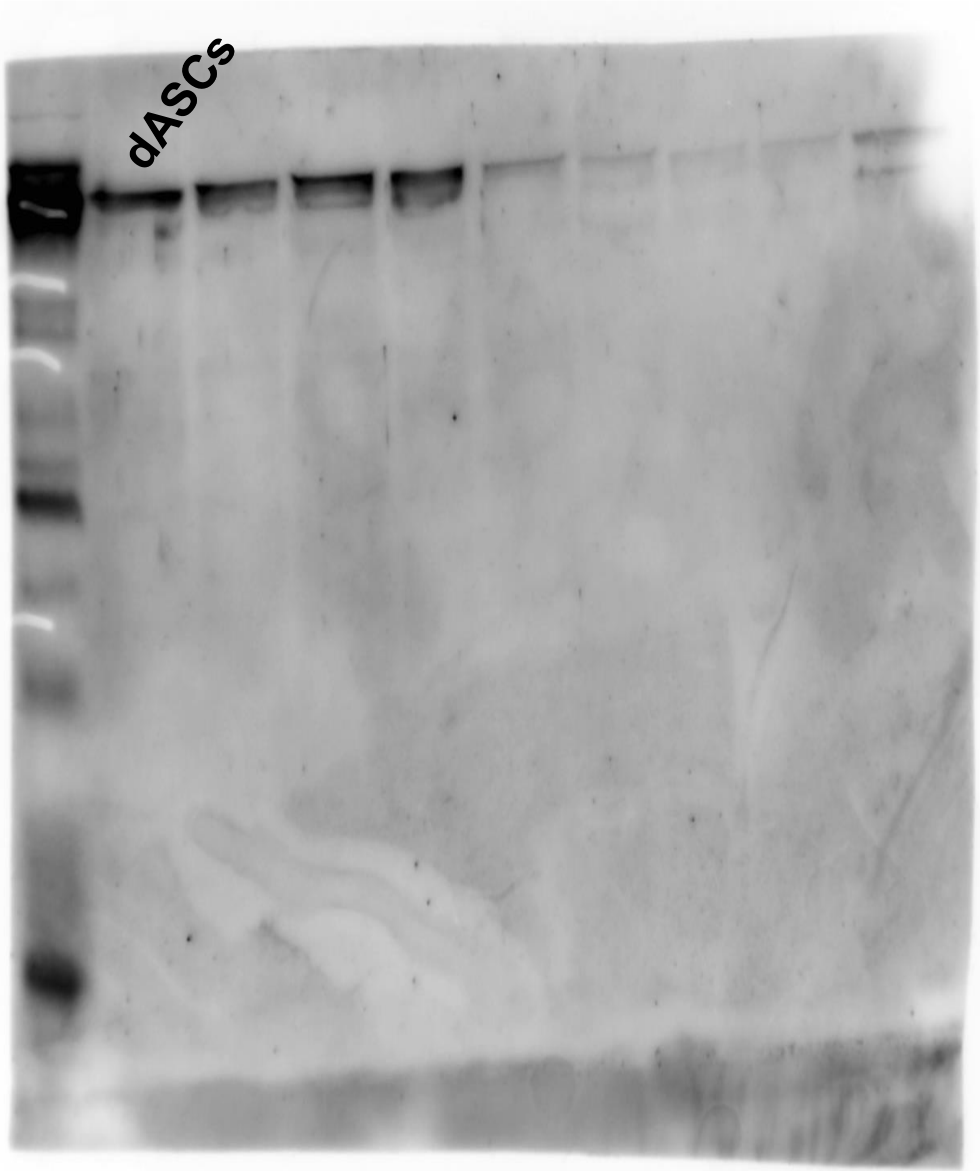

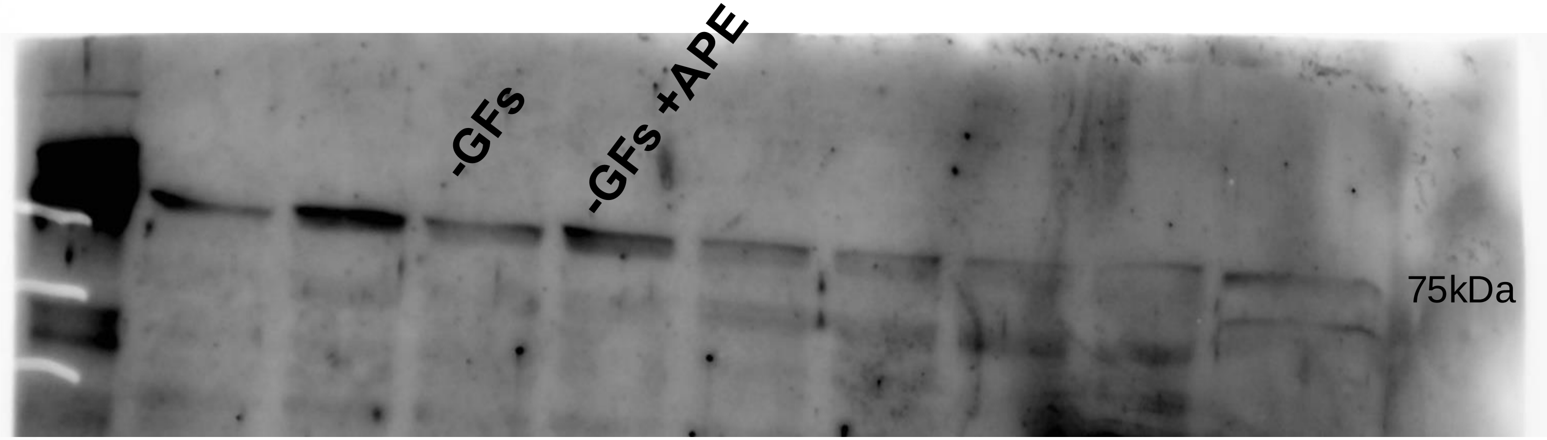

BDNF

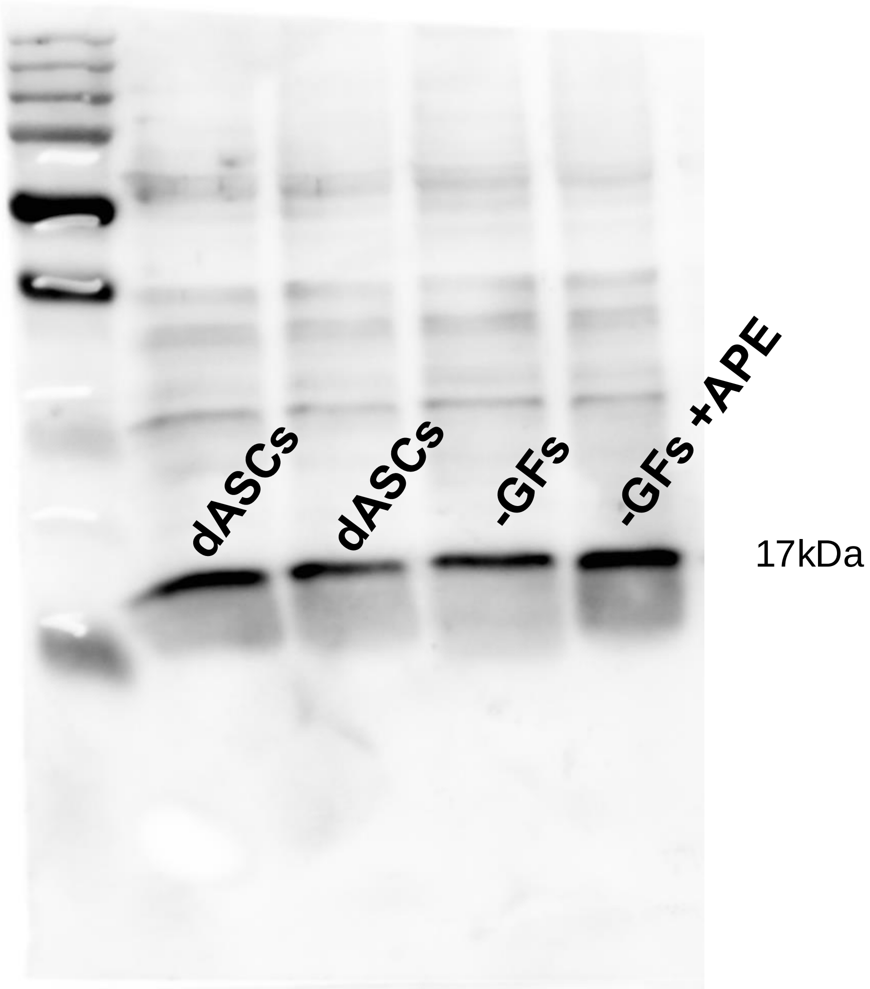

GAPDH

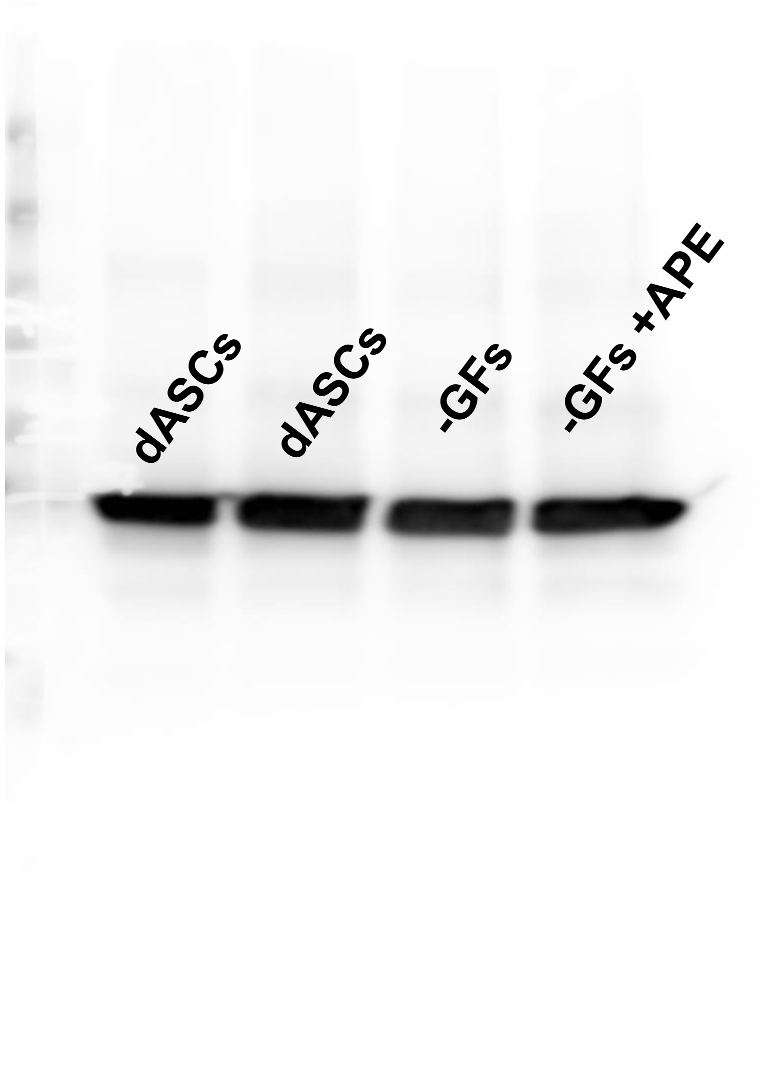

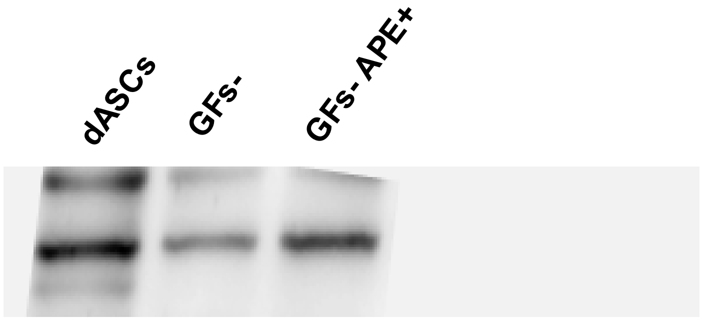

NGF
